# Modelling the impact of universal influenza vaccines on seasonal influenza with different subtypes

**DOI:** 10.1017/S0950268821002284

**Published:** 2021-11-02

**Authors:** Rui Li, Qian Li, Yiming Liu, Mingwang Shen, Lei Zhang, Guihua Zhuang

**Affiliations:** 1 China-Australia Joint Research Center for Infectious Diseases, School of Public Health, Xi'an Jiaotong University Health Science Center, Xi'an, Shaanxi 710061, China; 2 Xi'an Center for Disease Control and Prevention, Xi'an, Shaanxi 710061, China; 3 Key Laboratory for Disease Prevention and Control and Health Promotion of Shaanxi Province, Xi'an, Shaanxi 710061, China; 4 Melbourne Sexual Health Centre, Alfred Health, Melbourne, Australia; 5 Central Clinical School, Faculty of Medicine, Nursing and Health Sciences, Monash University, Melbourne, VIC, Australia; 6 Department of Epidemiology and Biostatistics, College of Public Health, Zhengzhou University, Zhengzhou 450001, Henan, China

**Keywords:** Seasonal influenza, subtypes, universal influenza vaccine, vaccination pattern, vaccine effectiveness and coverage

## Abstract

Several candidates of universal influenza vaccine (UIV) have entered phase III clinical trials, which are expected to improve the willingness and coverage of the population substantially. The impact of UIV on the seasonal influenza epidemic in low influenza vaccination coverage regions like China remains unclear. We proposed a new compartmental model involving the transmission of different influenza subtypes to evaluate the effects of UIV. We calibrated the model by weekly surveillance data of influenza in Xi'an City, Shaanxi Province, China, during 2010/11–2018/19 influenza seasons. We calculated the percentage of averted infections under 2-month (September to October) and 6-month (September to the next February) vaccination patterns with varied UIV effectiveness and coverage in each influenza season, compared with no UIV scenario. A total of 195 766 influenza-like illness (ILI) cases were reported during the nine influenza seasons (2010/11–2018/19), of which the highest ILI cases were among age group 0–4 (59.60%) years old, followed by 5–14 (25.22%), 25–59 (8.19%), 15–24 (3.75%) and ⩾60 (3.37%) years old. The influenza-positive rate for all age groups among ILI cases was 17.51%, which is highest among 5–14 (23.75%) age group and followed by 25–59 (16.44%), 15–24 (16.42%), 0–4 (14.66%) and ⩾60 (13.98%) age groups, respectively. Our model showed that UIV might greatly avert influenza infections irrespective of subtypes in each influenza season. For example, in the 2018/19 influenza season, 2-month vaccination pattern with low UIV effectiveness (50%) and coverage (10%), and high UIV effectiveness (75%) and coverage (30%) could avert 41.6% (95% CI 27.8–55.4%) and 83.4% (80.9–85.9%) of influenza infections, respectively; 6-month vaccination pattern with low and high UIV effectiveness and coverage could avert 32.0% (15.9–48.2%) and 74.2% (69.7–78.7%) of influenza infections, respectively. It would need 11.4% (7.9–15.0%) of coverage to reduce half of the influenza infections for 2-month vaccination pattern with low UIV effectiveness and 8.5% (5.0–11.2%) of coverage with high UIV effectiveness, while it would need 15.5% (8.9–20.7%) of coverage for 6-month vaccination pattern with low UIV effectiveness and 11.2% (6.5–15.0%) of coverage with high UIV effectiveness. We conclude that UIV could significantly reduce the influenza infections even for low UIV effectiveness and coverage. The 2-month vaccination pattern could avert more influenza infections than the 6-month vaccination pattern irrespective of influenza subtype and UIV effectiveness and coverage.

## Introduction

Influenza can cause severe diseases and economic burdens in a population. The World Health Organization (WHO) estimated that seasonal influenza infects a billion cases (3–5 million severe cases) and 290 000–650 000 respiratory deaths worldwide every year [1, 2]. In China, the national reported influenza infections in 2019 were more than 3.5 million, and the estimated annual economic burden related to influenza was 26.4 billion Chinese Yuan (CNY) [3, 4]. Influenza vaccination may substantially reduce the burden of influenza in a population, but the vaccination coverage varied largely across countries worldwide [5–8]. Developed countries, such as the USA and European member countries, are able to vaccinate half of their population (47.1–51.8% [9, 10]). In comparison, vaccination coverage in China is almost 30 times lower (1.5–2.2% [11]). This is largely due to insufficient production of influenza vaccine in China [12] and a low public perception towards the vaccine and its effectiveness in its population [13–15].

Universal influenza vaccines (UIVs) would induce more broad protection across different subtypes and durable immunity, by targeting the conserved epitopes and regions of influenza virus or stimulating cross-reactive T-cell responses, rather than driving antibody responses to the variable hemagglutinin head-like seasonal influenza vaccines [16, 17]. UIVs hold promise for expanding vaccination coverage. In February 2018, the National Institute of Allergy and Infectious Disease (NIAID) in the USA released its Universal Influenza Vaccine Strategic Plan to drive UIVs development and design next-generation influenza vaccines [18]. The USA, WHO and many other countries (European Union (EU), India, Australia, etc.) have approved more than billions of dollars to develop UIVs and there are currently three UIVs in phase III clinical trials [18–23]. An effective UIV was considered to provide robust, long-lasting and high effective protection against multiple subtypes of influenza [24], which made influenza vaccination more acceptable among the population without motivating people for annual vaccination [25]. Moreover, new egg-free production methods for UIVs would be applied so that sufficient vaccine doses can be produced in time [26–28].

Previous modelling studies have explored the impact of UIVs on public health benefits and social-economic in some high-income countries. DePasse *et al*. [29] used an agent-based model to examine the long-term effect of a UIV for the population aged 18–49 years in the USA and found that vaccination for the population aged 18–49 years would bring benefits to themselves directly and to children (<18 years old) and older (>49 years old) adults indirectly. France *et al*. [30] developed a Markov model to estimate the effect of a UIV compared with seasonal vaccines in hypothetical cohorts older than 65 years old in the USA and found that the UIV could be favoured if its effectiveness is comparable or better than a standard-dose vaccine. Sah *et al*. [31] developed a subtype/type- and age-specific dynamic transmission model of influenza based on data from 2010/11 to 2018/19 influenza seasons in the USA and estimated full use UIV replacement of seasonal vaccines was projected to prevent 17 million influenza infections, 251 000 hospitalisations, 19 500 deaths and $3.5 billion in direct health care costs per year. However, the possible impact of UIVs on influenza disease burden in low influenza vaccination coverage regions like China remains unclear.

In this paper, we proposed a new compartmental model to describe the transmission dynamics of seasonal influenza with different influenza subtypes. We calibrated the model by weekly surveillance data of influenza in Xi'an City, Shaanxi Province, China, during 2010/11–2018/19 influenza seasons. We aimed to evaluate the UIV effects by calculating the percentage of averted infections under two vaccination patterns based on WHO's recommendation [32], i.e. vaccinated in the first 2 months (September and October, 2-month pattern), or during half of the influenza season (September to the next February, 6-month pattern) with varied vaccination effectiveness and coverage in each influenza season, compared with no UIV scenario. Our results would provide insights into the impact of UIV and the optimal vaccination pattern in the less-developed areas.

## Methods

### Surveillance data and preprocessing

**Fig. 1.**
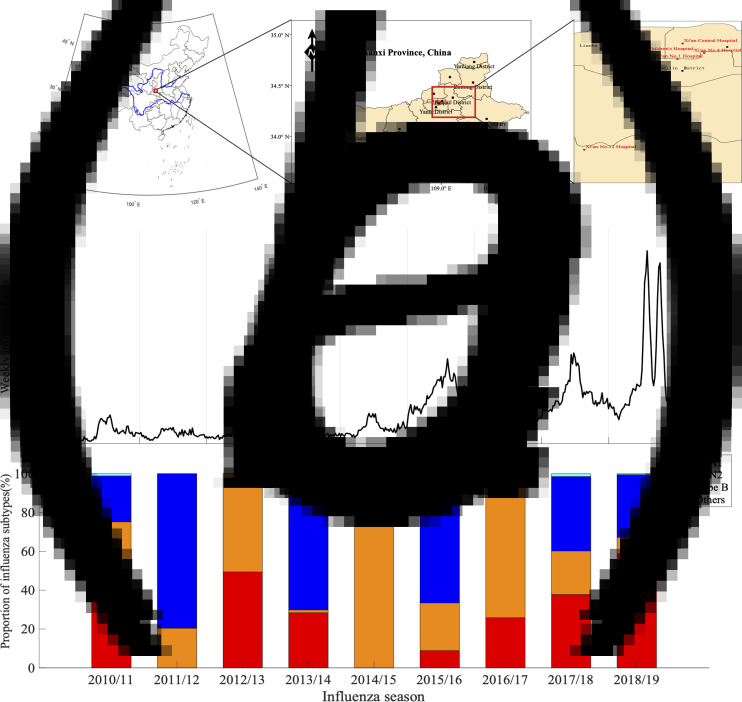
Time series of influenza surveillance data in Xi'an, China between 2010/11 and 2018/19 influenza seasons. (a) Map of China, marking Xi'an city (red rectangle in the left sub-figure), showing five influenza sentinel hospitals (red dot in the middle and right sub-figure). (b) The weekly time series of influenza infections. (c) The seasonal proportion of influenza subtypes in the positive influenza infections of ILIs.

We obtained the weekly time series of influenza data from week 27, 2010 to week 26, 2019 in Xi'an city, which is a temperate climate city in Northwest China (Fig. 1a) with about 10.2 million residents. The influenza sentinel hospitals and network laboratory, established by the Chinese National Influenza Center, aim to conduct influenza surveillance by reporting weekly influenza-like illness (ILI) cases and detecting the sampled specimens. Xi'an has five influenza sentinel monitoring hospitals (Xi'an Children's Hospital, Xi'an Central Hospital, Xi'an No.1 Hospital, Xi'an No.4 Hospital, Xi'an No.12 Hospital) and one influenza network laboratory (laboratory biosafety II or above and qualified to identify influenza subtypes and virus isolation), dispersed through four densely populated districts. Each week, the sentinel hospitals would use an Internet-based platform to report number of ILI cases (defined as patients whose body temperature ⩾38 °C with either cough or sore throat), divided into five age groups (i.e. 0–4, 5–14, 15–24, 25–59 and ⩾60 years). Moreover, every sentinel hospital was required to provide influenza network laboratory with 20 specimens (5–15 specimens before 2014 [33, 34]) of nasopharyngeal or throat swab per month (from April to September), and same specimens per week (from October to next March). The specimens of ILIs were collected from patients within 3 days of the appearance of symptoms and have not received any antiviral treatment, and then were transported in viral transport media at 4 °C to influenza surveillance network laboratory to isolate and identify influenza virus and subtypes [34]. In our model, we define week 27 to next week 26 as the whole influenza season. For example, week 27, 2010 to week 26, 2011 was denoted as the 2010/11 influenza season.

We obtained the weekly influenza infections by processing ILI cases and their influenza-positive rate in different age groups (Fig. 1b). The number of reported ILI cases and influenza-positive rates *s* differ in five age groups in the nine influenza seasons (see Appendix Fig. S1). Denote *c*_*i*,*t*_ as the weekly reported ILI cases in *i*-th (*i* = 1, 2, …5) age group at time *t*, and *θ*_*i*_ as the influenza-positive rate in *i*-th (*i* = 1, 2, …5) age group during each influenza season (July to next June, about 1700–3700 specimens were tested in each influenza season) (see Appendix Fig. S1C). The total weekly influenza infections *C*_*t*_ were calculated as follows.1
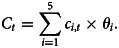


### Model construction

**Fig. 2.**
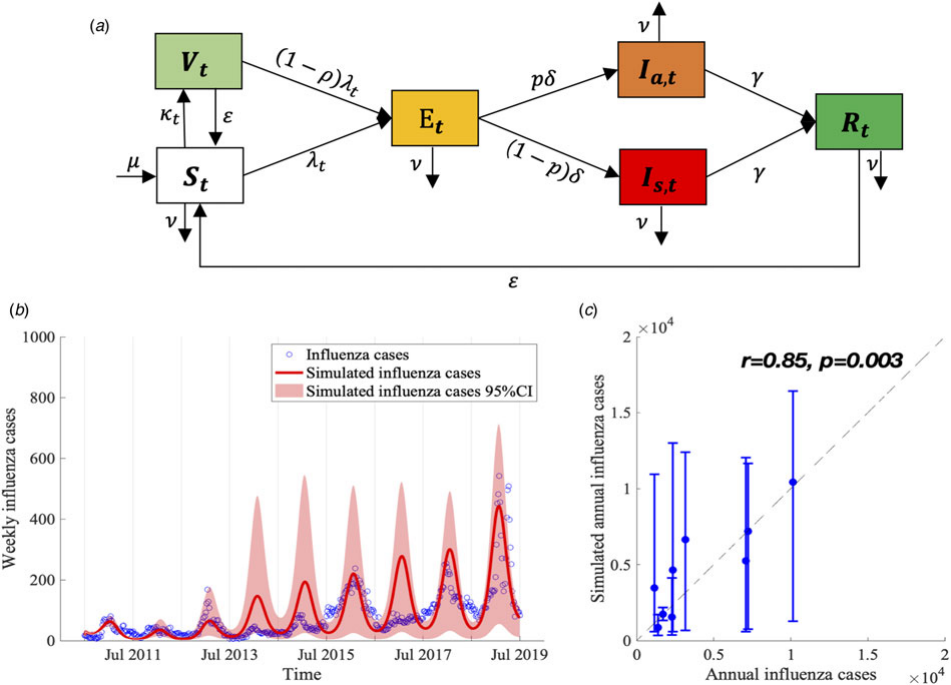
Flow chart of influenza transmission model and best model fit. (a) The total population is divided into six compartments (*S*_*t*_, *V*_*t*_, *E*_*t*_, *I*_*s*,*t*_, *I*_*a*,*t*_ and *R*_*t*_ denote the number of susceptible, vaccinated, exposed but not yet infectious, infectious with and without symptoms, and recovered individuals at time *t*, respectively). The force of infection for susceptible is denoted as *λ*_*t*_, which involves influenza subtypes of different seasons. We assume that *λ*_*t*_ varies periodically as a sine function annually. More details are described in Materials and methods section. (b) Model calibration and data fitting based on weekly time series influenza infections between 2010/11 and 2018/19 influenza seasons. (c) Model validation based on the Pearson correlation coefficient of simulated and observed annual influenza infections.

We proposed a compartmental model to describe the transmission of seasonal influenza with different influenza subtypes (Fig. 2a). The population was divided into six compartments: susceptible individuals (*S*_*t*_), vaccinated individuals (*V*_*t*_), latent infections but not yet infectious (*E*_*t*_), infectious individuals with (*I*_*s*,*t*_) and without (*I*_*a*,*t*_) symptoms, and recovered individuals (*R*_*t*_). The model is given as follows:2
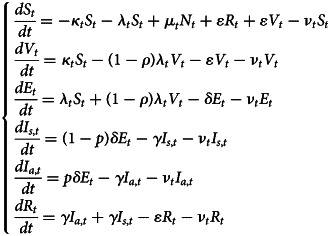
where *λ*_*t*_ is the force of influenza infection at time *t* (i.e. the probability of susceptible population being infected by asymptomatic or asymptomatic infected person) and it is given by3
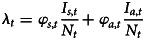

Here *φ*_*s*,*t*_, *φ*_*a*,*t*_ are the transmission rates when contacting with symptomatic and asymptomatic infections, respectively, which are assumed to change periodically in different influenza seasons based on the periodic characteristics of the influenza epidemic. We used sinusoidal function to express *φ*_*s*,*t*_, *φ*_*a*,*t*_ as follows:4
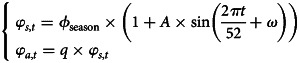
where *ϕ*_season_ represents the baseline transmission rate decided by the transmission rate of different influenza subtypes and their proportion in each influenza season, given by5
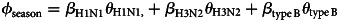


Here *β*_H1N1_, *β*_H3N2_, *β*_type B_ are the transmission rates of H1N1, H3N2 and B type, respectively, while *θ*_H1N1_, *θ*_H3N2_ and *θ*_type B_ are the proportion of these subtypes in each influenza season (Fig. 1c). *q* is the relative transmissibility for asymptomatic infections compared with symptomatic infections. *A* (⩽1) is the seasonal amplitude, and *ω* is the phase shift in the sinusoidal function. *p* is the fraction of asymptomatic infection. *μ*_*t*_ denotes the natural birth rate and *ν*_*t*_ denotes the natural death rate. Denote 1/*δ*, 1/*γ* and 1/ɛ as the average period of latency, recovery and immunity protection, respectively. *κ*_*t*_ represents the vaccination rate of UIV for susceptible individuals at time *t* and *ρ* denotes the effectiveness of UIV. Our model did not consider the effects of seasonal influenza vaccine due to very low seasonal influenza vaccine coverage (~2% in China, <0.5% in Xi'an).

### Parameter estimation and model calibration

We obtained the following four parameters from the published literatures. The mean incubation time for influenza is 2 (1–4) days (1/*δ* = 2) [35]. The fraction of asymptomatic infection was chosen as *P* = 40% (30–50%) [36]. The natural birth rate (*μ*_*t*_) and death rate (*ν*_*t*_) were obtained from the Xi'an Bureau of Statistics [37] (see Appendix Table S2). The other parameters were estimated by fitting the model (equations (2)–(5)) to the weekly time series of influenza cases from week 27, 2010 to week 26, 2019 (Fig. 2b) using the Nonlinear Least Square (NLS) method, which minimised the residual error of model-simulated and reported influenza infections ((1 − *p*)*δE*_*t*_ − *C*_*t*_)^2^. These initial estimates are used as prior information for carrying out the Bayesian Markov Chain Monte Carlo (MCMC) procedure with an adaptive Metropolis-Hastings (M-H) algorithm and post estimates can be obtained [38]. The algorithm was run for 10 000 iterations with a burn-in (some iterations at the beginning of an MCMC run are thrown away) of 3000 iterations, and we used the rest 7000 iterations to derive the mean value and 95% confidence intervals (CI) of parameters as shown in Table 1. We conducted Latin Hypercube Sampling (LHS) 1000 times [39] in estimated parameters and its 95% CI to run the dynamic model procedure and obtain the 95% CI of influenza incidence cases.

**Table 1.** Prior information of estimated parameters based on references or assumptions and its' post estimate values using MCMC methods

Symbol	Description	Prior information (range)	Sources	Post estimate (95% CI)
*β*_H1N1_	The transmission rate of H1N1 influenza subtype	1.4 (1–1.5)	Assumed	1.3663 (1.3610–1.3704)
*β*_H3N2_	The transmission rate of H3N2 influenza subtype	1.3 (1–1.5)	Assumed	1.3074 (1.2965–1.3135)
*β*_*t*ype B_	The transmission rate of Type B influenza subtype	1.4 (1–1.5)	Assumed	1.3157 (1.3103–1.3340)
1/*γ*	The period of recovery/weeks	7.7 (6.3–8.4)	[35]	6.5840 (6.5411–6.6115)
1/*ɛ*	The period of immunity protection/years	1.92 (0.38–19.2)	[44]	0.7588 (0.6847–0.9934)
*q*	The relative transmissibility for asymptomatic infections compared with symptomatic infections	0.6 (0.1–0.9)	[36]	0.5522 (0.5393–0.5666)
*A*	The seasonal amplitude in the sinusoidal function	0.16 (0.05–0.3)	Assumed	0.1472 (0.1420–0.1560)
*ω*	The phase shift in the sinusoidal function	5.8 (0.01–6.28)	Assumed	5.7379 (5.7162–5.7597)
*S*_0_	Initial number of susceptible individuals	550 000 (100 000–800 000)	Assumed^a^	564 009 (558 744–568 667)
*I*_*a*, 0_	Initial number of asymptomatic infected individuals	90 (1–200)	Assumed	94.03 (89.58–99.00)
*I*_*s*, 0_	Initial number of symptomatic infected individuals	10 (1–200)	Assumed	1.79 (1.72–1.95)
*R*_0_	Initial number of recovered individuals	60 000 (50 000–90 000)	Assumed	63 137 (62 469–63 897)

a The average population size served by each hospital is about 110 000 (total 100 hospitals in Xi'an city with population size 11 million), so we assumed the population size covered by 5 sentinel hospitals was about 550 000.

We calculated the Pearson correlation coefficient *r* and coefficient of determination *R^2^* [40, 41] to evaluate the goodness of fit between model-simulated and reported influenza infections in each influenza season (Fig. 2c). All the procedures and analyses were implemented by MATLAB R 2019b.

### Construction of scenarios

We considered two vaccination patterns about the vaccination time based on WHO's recommendations [32]. First, WHO recommends that the ideal time to implement the influenza vaccination is in fall, before the influenza season begins (November in the northern hemisphere), so we choose the time of vaccination as between September and October, i.e. 2-month pattern. Second, WHO also recommends that people could be vaccinated at any time during the influenza season to prevent more infections, so we choose another vaccination pattern that people can be vaccinated between September and the following February, i.e. 6-month pattern, which is closer to the current situation of real-world influenza vaccination.

Based on NIAID's influenza research programme [18] and the average effectiveness of the seasonal influenza vaccines across different subtypes [7], we defined the UIV with a 75% effectiveness as high effectiveness and a 50% effectiveness as low effectiveness. The current vaccine coverage in China is as low as 1.5–2.2% [11] and we consider it would probably increase to 10% after UIV rollout. Thus, we defined a 10% coverage as low coverage and a 30% coverage as high coverage.

**Fig. 3.**
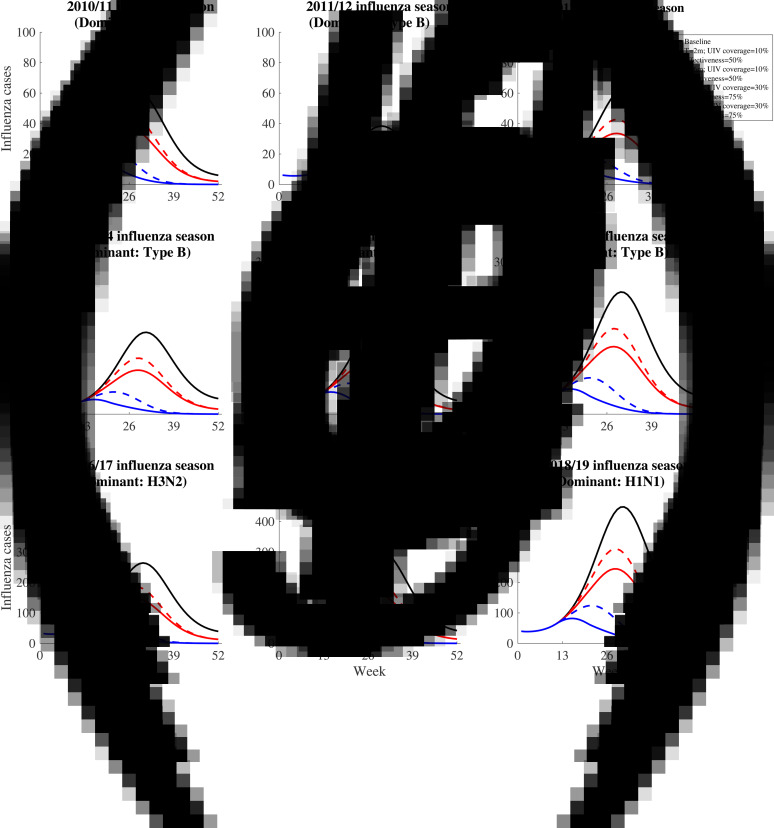
The simulated influenza infections for five constructed scenarios between 2010/11 and 2018/19 influenza season. The solid black line means influenza infections in the no vaccine scenario. The dotted red line means influenza infections in the 6-month vaccination pattern with low UIV coverage rate and effectiveness scenario. The solid red line means influenza infections in the 2-month vaccination pattern with low UIV coverage rate and effectiveness scenario. The dotted blue line means influenza infections in the 6-month vaccination pattern with high UIV rate and effectiveness scenario. The solid blue line means influenza infections in the 2-month vaccination with high UIV coverage rate and effectiveness scenario.

We projected the number of influenza infections in each influenza season under the following five scenarios (Fig. 3): (1) the no UIV scenario (baseline scenario); (2) the 2-month vaccination pattern with low UIV coverage rate (10%) and effectiveness (50%) scenario; (3) the 6-month vaccination pattern with low UIV coverage rate (10%) and effectiveness (50%) scenario; (4) the 2-month vaccination pattern with high UIV coverage rate (30%) and effectiveness (75%) scenario; (5) the 6-month vaccination pattern with high UIV coverage rate (30%) and effectiveness (75%) scenario. Previous study has reported the time window of antibody response is about 2 weeks after vaccination [42], so we assumed there was 2 weeks delay of immunity protection of vaccinated individuals.

### Sensitivity analysis

**Fig. 4.**
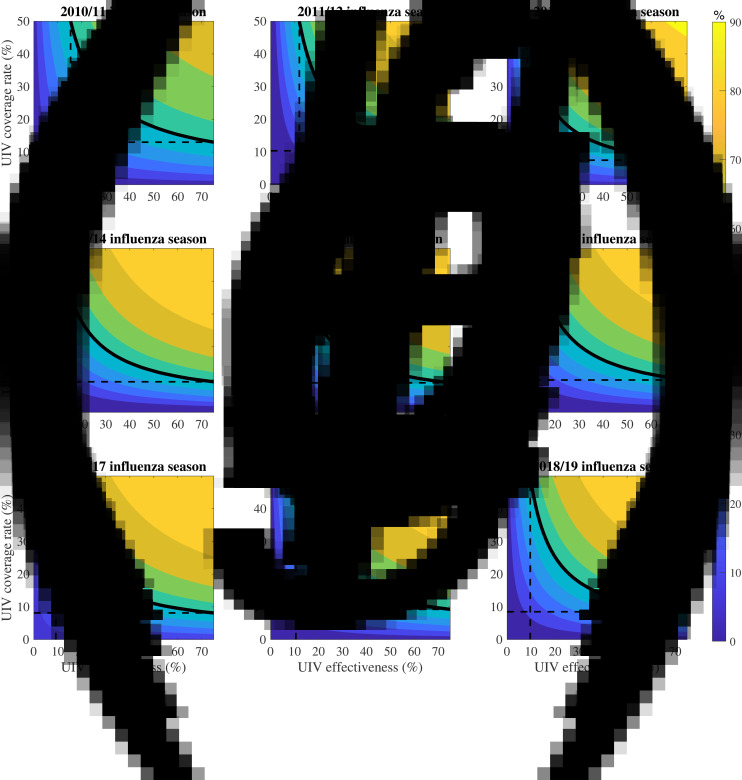
Contour plots about the percentage of averted infections as a function of universal influenza vaccine (UIV) coverage and effectiveness with 2-month vaccination pattern from 2010/11 to 2018/19 influenza seasons. The solid black isoclines indicate the threshold that the percentage of averted infections is 50% which is just for show. The dashed black lines correspond to the minimal vaccine effectiveness and vaccine coverage rate when the percentage of averted infections is 50%.

We expended the range of UIV coverage rate (0–50%) and effectiveness (0–75%) to perform sensitivity analysis (Fig. 4). Under the 2-month vaccination pattern, we calculated the percentage of averted influenza infections with varied UIV effectiveness and coverage in each influenza season, and plotted them as a function of UIV effectiveness and coverage. We also gave a special example that defined 50% of averted influenza infections as a threshold to evaluate the effects of varied UIV effectiveness and coverage. A similar plot for the 6-month vaccination pattern was shown in Appendix Fig. S2.

## Results

### General characteristics of the influenza epidemic

The proportion and influenza-positive rates of ILI cases differ in five age groups in the nine influenza seasons (2010/11–2018/19). During the nine seasons, a total of 195 766 ILI cases were reported by the five sentinel hospitals in Xi'an city, of which the highest ILI cases were among age group 0–4 (59.60%) years old, followed by 5–14 (25.22%), 25–59 (8.19%), 15–24 (3.75%) and ⩾60 (3.37%) years old (Appendix Fig. S1B). The influenza-positive rate for all age groups among ILI cases was 17.51%, which is highest among 5–14 (23.75%) age group and followed by 25–59 (16.44%), 15–24 (16.42%), 0–4 (14.66%) and ⩾60 (13.98%) age groups, respectively (Appendix Fig. S1C).

The proportion of influenza subtypes largely varied in different seasons and no one subtype could dominate for two consecutive influenza seasons (Fig. 1c). H3N2 subtype dominated in 2012/13 (50.60%), 2014/15 (82.73%), 2016/17 (73.23%) influenza seasons, while Type B subtype dominated in 2011/12 (79.59%), 2013/14 (69.78%), 2015/16 (66.43%) and 2017/18 (38.36%) influenza seasons.

Our model demonstrated a good calibration to the influenza infections, demonstrated by a significant positive correlation between the model-simulated cases and reported influenza infections (Fig. 2c, *r* = 0.853 and *R^2^* = 0.728, *P* = 0.003).

### Impact of universal influenza vaccination

Universal influenza vaccination might avert a substantial number of influenza infections in the four vaccination scenarios (scenario 2–5) irrespective of subtypes in each influenza season, compared with no UIV scenario (scenario 1) (Fig. 3). Two-month vaccination pattern with high UIV coverage and effectiveness (scenario 4) can avert the most influenza infections. The benefits of UIV vaccination are similarly huge in all seasons and we can recognise the whole through a single season. For example, in the 2018/19 influenza season, there are 10 413 (95% CI 1285–16 429) influenza infections in no UIV scenario; 2-month vaccination pattern with low and high UIV coverage and effectiveness could avert 41.6% (95% CI 27.8–55.4%) and 83.4% (80.9–85.9%) of influenza infections, respectively; 6-month vaccination pattern with low and high UIV effectiveness and coverage could avert 32.0% (15.9–48.2%) and 74.2% (69.7–78.7%) of influenza infections, respectively.

For the same UIV coverage and effectiveness, 2-month vaccination pattern could have the lower peak size and earlier peak time than 6-month vaccination pattern. Similarly, for the same pattern of vaccination, high UIV coverage and effectiveness could have the lower peak size and earlier peak time than low UIV coverage and effectiveness. For example, in the 2018/19 influenza season, the peak size of infections was 448 and the peak time was 31^st^ week in no UIV scenario; 2-month vaccination pattern with low and high UIV coverage and effectiveness could reduce the peak size to 245 (45.3%) and 82 (81.7%) influenza infections and bring forward the peak time to 28^th^ and 16^th^ week; 6-month vaccination pattern with low and high UIV coverage and effectiveness could reduce the peak size to 310 (30.8%) and 124 (72.3%) influenza infections and bring forward the peak time to 29^th^ and 21^st^ week.

### Sensitivity analysis

Greater UIV effectiveness and/or coverage rate would avert more percentage of influenza infections (Fig. 4). For example, in the 2018/19 influenza season, if the UIV effectiveness increased from 50% to 75% with 30% of UIV coverage unchanged, the percentage of averted influenza infections will increase from 75.1% (70.5–79.7%) to 83.4% (80.9–85.9%). If the UIV coverage rate increased from 30% to 50% with 75% of UIV effectiveness, the percentage of averted influenza infections compared with no UIV will increase from 83.4% (80.9–85.9%) to 89.6% (88.4–90.6%).

Larger UIV effectiveness would need lower coverage to avert 50% of influenza infections (Fig. 4). For example, in the 2018/19 influenza season, it would need 11.4% (7.9–15.0%) of coverage to reduce half of the influenza infections for 2-month vaccination pattern with low UIV effectiveness (50%) and 8.5% (5.0–11.2%) of coverage with high UIV effectiveness (75%), while it would need 15.5% (8.9–20.7%) of coverage for 6-month vaccination pattern with low UIV effectiveness and 11.2% (6.5–15.0%) of coverage with high UIV effectiveness (Fig. S2).

## Discussion

To the best of our knowledge, this is the first study to explore the population impact of UIV in China. Our model involved the percentage of different influenza subtypes in the transmission rate, which was not considered in the previous studies [29–31]. We found that UIV can significantly reduce the influenza infections in different influenza seasons with different subtypes. The larger effectiveness and coverage, the more averted influenza infections. Moreover, the 2-month vaccination pattern prior to the influenza season reduces more influenza infections than the 6-month vaccination pattern, for the same UIV effectiveness and coverage. This provides further theoretical support for the WHO's recommendation that vaccination should be completed by the end of October in the northern hemisphere [32]. Therefore, the centres of disease control and prevention should remind the public to get vaccinated within 2 months before the influenza season begins, especially in those regions with limited vaccine supply [12].

Our finding demonstrates that both the highly effective vaccines and the concentrated vaccination (the 2-month vaccination pattern) can improve the impact of UIV on decreasing the influenza incidence for the same coverage. We estimated that a UIV with high effectiveness (75%) could avert extra ~10% of influenza infections than a low one (50%) with the same UIV coverage (30%). The concentrated vaccination could also avert extra ~10% influenza infections than the 6-month vaccination pattern, irrespective of the high/low UIV coverage and effectiveness. This informed that changing the vaccination pattern would also be beneficial to control the transmission of influenza.

Our finding demonstrates that expanding UIV coverage brings enormous benefits for averting infections, but its effects become saturated with the growth of UIV coverage. Particularly, improving the coverage from 0% to 10% would avert ~50% infections, whereas improving the coverage from 30% to 50% would only avert extra ~10% infections. For China with only ~2% coverage of seasonal influenza vaccine before [11], even slight increase in UIV coverage to 10% can reduce greatly the influenza burden once UIV was rolled out.

Our study has some limitations. First, we did not model the transmission dynamics for different age groups. This is because the tested samples are so scarce that the surveillance data on influenza-positive rate and proportion of subtype by each age group per week are not available (too sparse). We combine the data in each age group together as a whole to obtain the total number of influenza infections for different subtypes. Second, our model only calculated the effect of the UIV in the short term (assuming the protection duration of UIV is the same as seasonal influenza vaccine conservatively) and did not further evaluate the impact of variation in the protection duration of UIV on the results. If its duration was longer, UIV can avert more influenza infections. Otherwise, if the immunity duration of UIV is shorter, the benefit of UIV may become lower. Third, we did not consider the superinfection and coinfection with multiple subtypes and we assumed the same UIV effectiveness for three influenza type/subtypes. This may be different from that the current seasonal influenza vaccine has poor effectiveness on the A/H3N2 subtype. If the UIV was less effective against H3N2, our model may overestimate the averted influenza infections during an H3N2-dominated influenza season. Finally, while our model is calibrated to the data in Xi'an city and the results may not be generalisable to other cities in China or other countries, our model can be applied to other settings to evaluate the vaccination strategies and the impact of UIV rollout on influenza infections [43].

In summary, UIV could significantly reduce the influenza infections even for low UIV effectiveness and coverage. The 2-month vaccination pattern averted more influenza infections than the 6-month vaccination pattern irrespective of influenza subtype and UIV effectiveness and coverage.

## Data Availability

The data that support the findings of this study are available from Xi'an Center for Disease Control and Prevention. Restrictions apply to the availability of these data, which were used under licence for this study. Data are available from the authors with the permission of Xi'an Center for Disease Control and Prevention.

## Supplementary material

For supplementary material accompanying this paper visit http://dx.doi.org/10.1017/S0950268821002284. click here to view supplementary material
