# Supplementary material for: Modelling the impact of universal influenza vaccines on seasonal influenza with different subtypes
Source: Epidemiol Infect. 2021 Nov 2;149:e253. doi: 10.1017/S0950268821002284 (PMC8697312; doi:10.1017/S0950268821002284)
Supplement: Supplementary file 1 [file S0950268821002284sup001.docx]

**Supplementary Appendix:**

**Modeling the Impact of Universal Influenza Vaccines on the seasonal influenza with different subtypes**

This is a supplementary document describing surveillance data details and model results. In section 1, we present more surveillance data details. In section 2, we provide more figures to support the results in the main text.

**1. Surveillance Data**

We obtained data of seasonal proportion of influenza subtypes from the 2010/11 to 2018/19 influenza seasons from influenza network laboratory (Xi 'an Center for Disease Control and Prevention) as shown in Table S1. The natural birth rate and death rate were followed from Xi’an Bureau of Statistics as shown in Table S2.

**Table S1.** **Seasonal proportion of influenza subtypes in 9 influenza seasons in Xi 'an, China(%)**

|  | 2010/11 | 2011/12 | 2012/13 | 2013/14 | 2014/15 | 2015/16 | 2016/17 | 2017/18 | 2018/19 |
| --- | --- | --- | --- | --- | --- | --- | --- | --- | --- |
| $\theta_{H1N1}$ | 49.13 | 0.00 | 49.40 | 28.36 | 0.00 | 8.86 | 25.81 | 37.77 | 58.77 |
| $\theta_{H3N2}$ | 26.01 | 20.41 | 50.60 | 1.49 | 82.73 | 24.59 | 73.23 | 22.45 | 8.47 |
| $\theta_{typeB}$ | 23.70 | 79.59 | 0.00 | 69.78 | 17.27 | 66.43 | 0.76 | 38.36 | 32.27 |
| Others | 1.16 | 0.00 | 0.00 | 0.37 | 0.00 | 0.12 | 0.19 | 1.43 | 0.49 |

**Table S2.** **The natural birth rate and death rate in 9 influenza seasons in Xi 'an, China(‰)**

| Season | Natural birth rate ($\mu_{t}$) | Natural death rate ($\nu_{t}$) | Season | Natural birth rate ($\mu_{t}$) | Natural death rate ($\nu_{t}$) |
| --- | --- | --- | --- | --- | --- |
| 2010/11 | 9.73 | 5.34 | 2015/16 | 10.15 | 5.51 |
| 2011/12 | 9.71 | 5.38 | 2016/17 | 11.54 | 5.40 |
| 2012/13 | 10.13 | 5.57 | 2017/18 | 12.62 | 5.42 |
| 2013/14 | 9.57 | 5.37 | 2018/19 | 12.47 | 5.48 |
| 2014/15 | 10.11 | 5.47 |  |  |  |

The per-capita benefits (averted infections per 10,000 doses vaccinations) in the 5 constructed scenarios of 9 influenza season are shown in Table S3. For the low coverage (10%) and effectiveness (50%), 2-month strategy can avert 57.8-63.3% more infections per 10,000 doses vaccinations than 6-month strategy, and 40.8-45.8% for the high coverage (30%) and effectiveness (75%).

**Table S3.** **The** **avert infections per 10,000 doses vaccinations for 5 constructed scenarios from 2010/11 to 2018/19 influenza season.**

| Influenza Season | Baseline | UIV cov=10%  eff=50%, T=2m | UIV cov=10%  eff=50%, T=6m | UIV cov=30%  eff=75%, T=2m | UIV cov=30%  eff=75%, T=6m |
| --- | --- | --- | --- | --- | --- |
| 2010/11 season |  |  |  |  |  |
| Vaccinated doses | 0 | 67142 | 84095 | 202178 | 257403 |
| Infected cases | 1705 | 1169 | 1314 | 559 | 730 |
| Averted cases | 0 | 536 | 391 | 1146 | 975 |
| Averted cases/Vaccinated cases (1/10,000 doses) | 0 | 80 | 46 | 57 | 38 |
| 2011/12 season |  |  |  |  |  |
| Vaccinated doses | 0 | 71025 | 88116 | 213808 | 269482 |
| Infected cases | 935 | 564 | 653 | 192 | 281 |
| Averted cases | 0 | 371 | 282 | 743 | 654 |
| Averted cases/Vaccinated cases (1/10,000 doses) | 0 | 52 | 32 | 35 | 24 |
| 2012/13 season |  |  |  |  |  |
| Vaccinated doses | 0 | 72335 | 89491 | 217735 | 273682 |
| Infected cases | 1484 | 835 | 978 | 228 | 358 |
| Averted cases | 0 | 649 | 506 | 1256 | 1126 |
| Averted cases/Vaccinated cases (1/10,000 doses) | 0 | 90 | 57 | 58 | 41 |
| 2013/14 season |  |  |  |  |  |
| Vaccinated doses | 0 | 72833 | 89986 | 219241 | 275317 |
| Infected cases | 3775 | 2155 | 2522 | 605 | 943 |
| Averted cases | 0 | 1620 | 1253 | 3170 | 2832 |
| Averted cases/Vaccinated cases (1/10,000 doses) | 0 | 222 | 139 | 145 | 103 |
| 2014/15 season |  |  |  |  |  |
| Vaccinated doses | 0 | 73021 | 90201 | 219818 | 276069 |
| Infected cases | 4566 | 2759 | 3198 | 913 | 1355 |
| Averted cases | 0 | 1807 | 1368 | 3653 | 3211 |
| Averted cases/Vaccinated cases (1/10,000 doses) | 0 | 247 | 152 | 166 | 116 |
| 2015/16 season |  |  |  |  |  |
| Vaccinated doses | 0 | 73252 | 90488 | 220518 | 276995 |
| Infected cases | 5709 | 3371 | 3916 | 1038 | 1574 |
| Averted cases | 0 | 2338 | 1793 | 4671 | 4135 |
| Averted cases/Vaccinated cases (1/10,000 doses) | 0 | 319 | 198 | 212 | 149 |
| 2016/17 season |  |  |  |  |  |
| Vaccinated doses | 0 | 73547 | 90850 | 221414 | 278190 |
| Infected cases | 6238 | 3731 | 4329 | 1183 | 1779 |
| Averted cases | 0 | 2507 | 1909 | 5055 | 4459 |
| Averted cases/Vaccinated cases (1/10,000 doses) | 0 | 341 | 210 | 228 | 160 |
| 2017/18 season |  |  |  |  |  |
| Vaccinated doses | 0 | 73881 | 91288 | 222428 | 279578 |
| Infected cases | 7170 | 4346 | 5032 | 1427 | 2124 |
| Averted cases | 0 | 2824 | 2138 | 5743 | 5046 |
| Averted cases/Vaccinated cases (1/10,000 doses) | 0 | 382 | 234 | 258 | 180 |
| 2018/19 season |  |  |  |  |  |
| Vaccinated doses | 0 | 74329 | 91749 | 223787 | 281196 |
| Infected cases | 10413 | 6069 | 7062 | 1726 | 2684 |
| Averted cases | 0 | 4320 | 3327 | 8663 | 7705 |
| Averted cases/Vaccinated cases (1/10,000 doses) | 0 | 581 | 363 | 387 | 274 |
|  |  |  |  |  |  |

**2. Effective reproduction number**

The effective reproduction number is calculated as follows [1, 2]:

$R_{e}(t)={eigenvalue}_{max}(FV^{-1})=\frac{\left[ S_{t}+\left( 1-\rho\right)V_{t} \right]\varphi_{s,t}}{N_{t}} \frac{\left( 1-p \right)\delta}{(\delta+\nu_{t})(\gamma+\nu_{t})}$+$\frac{q\left[ S_{t}+\left( 1-\rho\right)V_{t} \right]\varphi_{s,t}}{N_{t}}$ $\frac{p\delta}{(\delta+\nu_{t})(\gamma+\nu_{t})}$

where F and V are given by

$F=\left( \begin{matrix} 0 & \frac{\left[ S_{t}+\left( 1-\rho\right)V_{t} \right]\varphi_{s,t}}{N_{t}} & \frac{q\left[ S_{t}+\left( 1-\rho\right)V_{t} \right]\varphi_{s,t}}{N_{t}} \\ 0 & 0 & 0 \\ 0 & 0 & 0 \end{matrix} \right)$, $V=\left( \begin{matrix} \delta+\nu_{t} & 0 & 0 \\ -\left( 1-p \right)\delta& (\gamma+\nu_{t}) & 0 \\ -p\delta& 0 & (\gamma+\nu_{t}) \end{matrix} \right)$,

and $\varphi_{s,t}$ and $\varphi_{a,t}$ are given by

$\begin{aligned} \varphi_{s,t}=\phi_{season}\times\left( 1+A\times\sin\left( \frac{2\pi t}{52}+\omega\right) \right) \\ \varphi_{a,t}=q\times\varphi_{s,t}. \end{aligned}$,

as shown in the main text. We plotted effective reproductive numbers in all scenarios of 9 influenza seasons in **Figure S3**.

**3.Supplementary Figures**

Figure S1 shows time series of ILIs surveillance data in Xi’an, China between 2010/11 to 2018/19 influenza season. Figure S1 A shows the weekly time series of ILIs in different age group. Figure S1 B shows the seasonal proportion in different age group of ILIs between. Figure S1 C shows the seasonal positive rate of ILIs in different age group.

Figure S2 shows that Greater vaccine effectiveness and/or coverage rate would lead to more averted influenza cases. For example, in the 2018/19 influenza season, if the vaccine effectiveness increased from 50% to 75% with 30% of vaccine coverage, the percentage of averted influenza infections will increase from 64.3% (57.3-71.4%) to 74.2% (69.7-78.7%). If the vaccine coverage rate increased from 30% to 50% with 75% of vaccine effectiveness, the percentage of averted influenza infections compared with no UIV will increase from 74.2% (69.7-78.7%) to 84.3% (82.2-86.4%).74.2% (69.7-78.7%). Larger vaccine effectiveness would need lower coverage to avert 50% of cases, it would need 15.5% (8.9-20.7%) of coverage to reduce half of the influenza cases with low vaccine effectiveness (50%) and 11.2% (6.5-15.0%) of coverage with high vaccine effectiveness (75%).

Figure S3 shows that UIV might largely reduce the effective reproductive numbers of influenza by reducing the proportion of the population that is susceptible in the four vaccination scenarios (scenario 2-5) irrespective of subtypes in each influenza season, compared with no UIV scenario (scenario 1). Higher levels of vaccine effectiveness or coverage will yield lower effective reproductive numbers and thus smaller epidemic sizes. Furthermore, if the vaccines are administered faster (the 2-month strategy), the effective reproductive number will drop below 1 faster, which will bring the epidemic to a halt earlier (explaining the earlier peak time) and thus result in fewer people getting infected after the epidemic has turned around (explaining the lower number of infected cases).

**Reference**

1. van den Driessche P, Watmough J. Reproduction numbers and sub-threshold endemic equilibria for compartmental models of disease transmission. Mathematical Biosciences **2002**; 180:29-48.

2. Cintrón-Arias A, Castillo-Chávez C, Bettencourt LM, Lloyd AL, Banks HT. The estimation of the effective reproductive number from disease outbreak data. Mathematical Biosciences and Engineering **2009**; 6:261-82.

**Figure S1. Time series of influenza surveillance data in Xi’an, China from 2010/11 to 2018/19 influenza season. (**A) The weekly time series of ILIs in different age group (B) The seasonal proportion of ILIs in different age group. (C) The seasonal influenza-positive rate of ILIs in different age group.

**Figure S2. Contour plots about the percentage of averted infections as a function of universal influenza vaccine (UIV) coverage and effectiveness with 6-month vaccination pattern from 2010/11 to 2018/19 influenza season.** The solid black isoclines indicate the threshold that the percentage of averted infections is 50%. The dashed black lines correspond to the minimal vaccine effectiveness and vaccine coverage rate when the percentage of averted infections is 50%.

**Figure S3. The effective reproductive numbers for 5 constructed scenarios from 2010/11 to 2018/19 influenza seasons.** The solid black line means influenza infections in the no UIV scenario. The dotted (solid) red line means influenza infections in the 6-month (2-month) vaccination pattern with low UIV coverage rate and effectiveness scenario. The dotted (solid) blue line means influenza infections in the 6-month (2-month) vaccination pattern with high UIV rate and effectiveness scenario. The magenta dotted line represents the effective reproductive number is equal to 1. The period when the effective reproductive number exceeds 1 is roughly correspond to the annual influenza season in Xi’an city (September to following February).
